# Seed2LP: seed inference in metabolic networks for reverse ecology applications

**DOI:** 10.1093/bioinformatics/btaf140

**Published:** 2025-03-31

**Authors:** Chabname Ghassemi Nedjad, Mathieu Bolteau, Lucas Bourneuf, Loïc Paulevé, Clémence Frioux

**Affiliations:** University of Bordeaux, CNRS, BordeauxINP, LaBRI, UMR 5800, Talence F-33400, France; Inria, University of Bordeaux, INRAE, Talence F-33400, France; Inria, University of Bordeaux, INRAE, Talence F-33400, France; Nantes Université, Ecole Centrale Nantes, CNRS, LS2N, UMR 6004, Nantes F-44000, France; Inria, Université de Rennes, CNRS, IRISA, UMR 6074, Rennes F-35000, France; CHRU Brest, Université de Bretagne Occidentale, Brest F-29000, France; University of Bordeaux, CNRS, BordeauxINP, LaBRI, UMR 5800, Talence F-33400, France; Inria, University of Bordeaux, INRAE, Talence F-33400, France

## Abstract

**Motivation:**

A challenging problem in microbiology is to determine nutritional requirements of microorganisms and culture them, especially for the microbial dark matter detected solely with culture-independent methods. The latter foster an increasing amount of genomic sequences that can be explored with reverse ecology approaches to raise hypotheses on the corresponding populations. Building upon genome-scale metabolic networks (GSMNs) obtained from genome annotations, metabolic models predict contextualized phenotypes using nutrient information.

**Results:**

We developed the tool *Seed2LP*, addressing the inverse problem of predicting source nutrients, or *seeds*, from a GSMN and a metabolic objective. The originality of Seed2LP is its hybrid model, combining a scalable and discrete Boolean approximation of metabolic activity, with the numerically accurate flux balance analysis (FBA). Seed inference is highly customizable, with multiple search and solving modes, exploring the search space of external and internal metabolites combinations. Application to a benchmark of 107 curated GSMNs highlights the usefulness of a logic modelling method over a graph-based approach to predict seeds, and the relevance of hybrid solving to satisfy FBA constraints. Focusing on the dependency between metabolism and environment, Seed2LP is a computational support contributing to address the multifactorial challenge of culturing possibly uncultured microorganisms.

**Availability and implementation:**

Seed2LP is available on https://github.com/bioasp/seed2lp.

## 1 Introduction

In the past decades, metagenomic sequencing had a profound impact on microbiology with substantial expansion of known genome collections (Nayfach *et al.* 2021, Nishimura and Yoshizawa 2022, Zeng *et al.* 2022). While the previously widespread paradigm that only 1% of microorganisms are culturable tends to be rejected (Martiny 2019), estimations state that a high proportion of archaea and bacteria remain uncultured across most biomes (Steen *et al.* 2019). The prevalence of microbial interactions in natural environments is a strong hypothesis underlying the difficulty to culture many taxa (Yan *et al.* 2023), and more and more studies pinpoint the role of metabolism in setting up these interactions (Pande and Kost 2017, Pacheco *et al.* 2019). While microbiology is essential to solve the problem of culturability, computational methods taking advantage of the availability of genomes coupled to simulation approaches can provide complementary hypotheses to tackle this issue.

Computational models of metabolism enable the prediction of metabolic activity in organisms, taking into account a description of environmental conditions and additional constraints. In practice, the information related to the biochemical reactions a microbe could catalyse is abstracted in genome-scale metabolic networks (GSMNs), that are in turn used in models for simulation, such as *flux balance analysis* (FBA) (Orth *et al.* 2010). GSMNs can be automatically or semi-automatically built starting from genomes (Gu *et al.* 2019), although it is unrealistic to expect a high-quality GSMN based on a fully automatic process (Karp *et al.* 2018): manual curation, expert knowledge and refinement are needed to reach a level of quality sufficient for quantitative simulations of the model (Thiele and Palsson 2010, Bernstein *et al.* 2021). As a result, it is much more difficult to model the metabolism of poorly studied organisms, that are sometimes not experimentally grown in pure culture and defined medium, and for which only a (possibly incomplete) genome is available. For these cases, quantitative models of metabolism are hardly applicable in first intention. Topological analysis of the graph structure of GSMNs and qualitative simulations are then a good trade-off to gain knowledge on possibly incomplete models (Biggs *et al.* 2015, Bosi *et al.* 2017) and improve them prior to quantitative simulations (Bernstein *et al.* 2019).

Using GSMNs to predict the growth environment of a species has previously got attention. Three categories of approaches can be distinguished: structural methods relying on the topology of the graph, reachability methods enabling a qualitative abstraction of metabolic activation and constraint-based models ensuring steady-state. In the first category, Borenstein *et al.* (2008) calculated the minimal subset of exogenously acquired metabolites, or *seeds*, using the decomposition of a directed simple GSMN graph into strongly connected components (SCC). An SCC without an incoming edge is a source component among which one seed can be identified. An implementation of these concepts was proposed in NetSeed (Carr and Borenstein 2012), then in an R package for reverse ecology (Cao *et al.* 2016), and reused in a Python implementation (Hamilton *et al.* 2017).

In the second category, Romero and Karp (2001) first introduced a notion of qualitative graph-based activation as a means to predict the set of compounds that can be produced in a GSMN, regardless of their quantity, solving a forward propagation problem, and the backtracking problem identifying precursor compounds to produce essential metabolites. The forward identification of activable pathways and producible metabolites in GSMNs was later included in the *network expansion* (NE) algorithm (Ebenhöh *et al.* 2004) as a means to decipher the *scope* of a GSMN starting from a set of seed metabolites, i.e. available nutrients. The *scope* encompasses all the metabolites that can be reached from the seeds, regardless their stoichiometry in reactions. In line with this Boolean abstraction of metabolic producibility, Handorf *et al.* (2008) developed a greedy algorithm for the reverse scope problem, i.e. identifying precursors or seeds for a GSMN, by iteratively reducing the set of seed metabolites. A logic programming implementation using answer-set programming (ASP) of reverse scope has also been proposed by Schaub and Thiele (2009). It must be noted that the complexity of the reverse scope problem is NP-complete even in acyclic graphs (Nikoloski *et al.* 2008, Liu *et al.* 2009, Damaschke 2015). As an alternative, Cottret *et al.* (2008) and Acuña *et al.* (2012) provide algorithms computing precursors of a GSMN, with the originality of taking into account self-regenerating cycles, thus being less stringent than NE and the scope towards the initiation of reachability from seeds.

Lastly, in the third category, constraint-based approaches involve mixed-integer linear programming (MILP) and linear programming (LP) to ensure flux steady state. The MENTO algorithm (Zarecki *et al.* 2014) calculates minimal environments for genome-scale metabolic models, ensuring production of biomass, although no implementation is currently available. If a set of exchange reactions is pre-defined in the model, COBRApy (Ebrahim *et al.* 2013) implements two approaches: solving an LP problem that minimizes their corresponding sum of flux, and an MILP formulation that minimizes the number of activated exchange reactions, such as in the EMAF approach (Santos *et al.* 2017). SASITA (Andrade *et al.* 2016) also solves an MILP problem identifying sets of precursors which enable target metabolite production from the GSMN’s hypergraph while constraining the system with the steady-state assumption.

Graph-based and constraint-based methods of the literature do not ensure compound NE reachability, while reverse scope-based methods relying on NE do not guarantee steady flux in the objective reactions. Moreover, in practice, constraint-based approaches typically identify cardinal-minimal sets of seeds among pre-defined media of exchange reactions, which limits their application to highly curated GSMNs for which such reactions are identified. Our aim is to tackle these shortcomings by combining reverse scope computation and FBA, and by allowing all metabolites to be considered as potential seeds, including internal ones. Thus, the resulting seeds enable both a steady-state flux in the objective reaction and the triggering of necessary reactions for reaching the objective reaction. Moreover, by relieving the necessity to pre-determine potential reaction exchanges, we extend the applicability of these methods to less curated GSMNs, yet with a challenge on the scalability.

In this paper, we propose and benchmark several logic-based solving algorithms for the inverse scope problem combined with FBA. Instead of minimizing the cardinality of the sets of identified seeds, our algorithms focus on the identification of *subset-minimal* sets of seeds. This offers a larger variety of solutions. For instance, if {A,B} and {B,C,D} are both subset-minimal solution sets of seeds, only the former is returned by cardinal minimization. Moreover, any non-minimal solution extends at least one subset-minimal solution. We tested the scalability and relevance of our approach on 107 high-quality GSMNs from the BiGG database (King *et al.* 2016) demonstrating that a qualitative approach based on knowledge representation and reasoning is a good trade-off between computational efficiency and quality of the predictions. We provide a Python library, Seed2LP, that implements the solving using logic programming and permits customizing the parameters of the problem to fit the most use-cases in environment prediction from metabolic networks.

## 2 Material and methods

### 2.1 Metabolic networks

A metabolic network is a collection R of reactions r of the form

$$\alpha_{1}M_{1}+\cdots +\alpha_{a}M_{a}↔\beta_{1}N_{1}+\cdots +\beta_{b}N_{b}$$

where $M_{1},\ldots ,M_{a}$ and $N_{1},\ldots ,N_{b}$ are metabolites, and $\alpha_{1},\ldots ,\alpha_{a}$ and $\beta_{1},\ldots ,\beta_{b}$ are stoichiometric coefficients. Each reaction r is associated with lower and upper flux bounds $\nu_{\min,r},\nu_{\max,r}\in R$. Whenever one of the bounds is null, the reaction is said to be irreversible.

### 2.2 Flux balance analysis

FBA is a widely used mathematical model that aims at predicting flux distributions under a quasi-steady-state assumption for intracellular metabolites (Orth *et al.* 2010). Taking into account additional thermodynamic constraints bounding the flux values for each reaction, FBA solves a linear optimization problem that maximizes (or minimizes) the flux in an objective reaction $r_{T}\in R$, usually the biomass reaction that models growth.

The FBA formulation relies on the *stoichiometric matrix* of a metabolic network, which is a matrix $X\in R^{\left|M|\times |R\right|}$, where rows are the metabolites M, and columns the reactions R. Given a metabolite $m_{i}\in M$ and a reaction $r_{j}\in R$, the matrix stoichiometric coefficient $X_{i,j}$ indicates the net gain in metabolite $m_{i}$ whenever applying reaction $r_{j}$ positively. To ease notations, we assume hereafter that all metabolites are internal, i.e. each of them is produced and consumed by at least one reaction. The FBA problem is then formulated as follows, with $\nu \in R^{\left|R\right|}$ a vector of the reaction fluxes:

(1a) $$\text{maximize}\;\nu_{r_{T}}$$

 

(1b) subject to X.ν=0

 

(1c) $$\nu_{\min}\leq \nu \leq \nu_{\max}$$

A widely used exchange format for metabolic networks is the Systems Biology Markup Language (SBML) (Hucka *et al.* 2018) that is used by most metabolic modelling tools. Among the latter, COBRApy (Ebrahim *et al.* 2013) solves the FBA problem (1) by taking into account data-driven physicochemical and biological constraints. Those can be represented in the form of reactions that hold a special status within the metabolic network as they represent boundaries of the modelled system. By construction, they are unbalanced pseudo-reactions, with the objective to add or remove a metabolite for modelling purposes. An exchange reaction $r_{ex}$ is a reversible reaction with no product, adding and removing an extracellular metabolite $m_{e}\in M$. It is usually of the form $m_{e}↔\emptyset$, with a flux value $\nu_{r_{ex}}$ having a negative lower bound and a positive upper bound. The extracellular medium composition that contains nutrients necessary for cell growth will typically be represented as a collection of exchange reactions. A demand reaction $r_{dm}$ is an irreversible reaction that consumes an intracellular or cytosolic metabolite $m_{c}\in M$: $m_{c}\to \emptyset$, its flux $\nu_{r_{dm}}$ having a null lower bound and a positive upper bound. Lastly, a sink $r_{sk}$ is a reversible reaction without products, adding and removing a cytosolic metabolite $m_{c}\in M$: $m_{c}↔\emptyset$ with a flux value $\nu_{r_{sk}}$ having a negative lower bound and a positive upper bound.

### 2.3 Network expansion

The NE algorithm (Ebenhöh *et al.* 2004) makes a Boolean approximation of metabolic activity from compounds available in the environment. Starting from a subset of metabolites S⊆M, called *seeds*, the algorithm determines a metabolic potential, called *scope*, by inferring the activation of a reaction from the availability of all its reactants, either from seeds or products of other previously activated reactions.

The scope of a metabolic network N can be computed iteratively from a set of seeds S as described in Equation (2) until it reaches a fixed point. To ease notations, we assume that reactions are irreversible, and write reactants(r) and products(r) the reactants and products of a reaction r.

(2) $$\begin{array}{c} S\mathrm{cope}(N,S)=\underset{i}{\cup }M_{i}\;\text{where}\;M_{0}=S\;\text{and}\;\\ M_{i+1}=M_{i}\cup \underset{r\in R:\mathrm{reactants}(r)\subseteq M_{i}}{\cup }\mathrm{products}(r) \end{array}$$

### 2.4 Answer-set programming

Answer-set programming (ASP) is a logic programming paradigm suitable for knowledge representation and reasoning (Baral 2003). It enables solving combinatorial problems on Boolean variables subject to logical constraints. An ASP program consists in a set of atoms, forming the knowledge base, and rules enabling to express deductions, choices, and constraints. Clingo is an ASP solver (Gebser *et al.* 2019) that was previously used in the context of metabolic modelling applications (Frioux *et al.* 2018, Thuillier *et al.* 2021) and can be hybridized to verify linear constraints with clingo-lpx (https://github.com/potassco/clingo-lpx), enabling implementing Boolean constraints such as those from NE (Schaub and Thiele 2009), and checking the satisfiability of linear constraints from FBA, as previously demonstrated in Frioux *et al.* (2019). This programming approach permits the implementation of optimizations such as subset minimal or minimal sets of solutions, and offers the possibility of implementing various solving heuristics for the exploration of the solution space by adding constraints and going back and forth with the solver.

## 3 Results

### 3.1 Contributed method

#### 3.1.1 Model-network reconciliation

The representation of a metabolic model in the SBML format can show inconsistencies between the description of the mathematical model and the structure of the network. As our method relies on both descriptions, it necessitates a normalization step to reconcile them, particularly in terms of reaction boundaries or reversibility.

**Figure 1.**
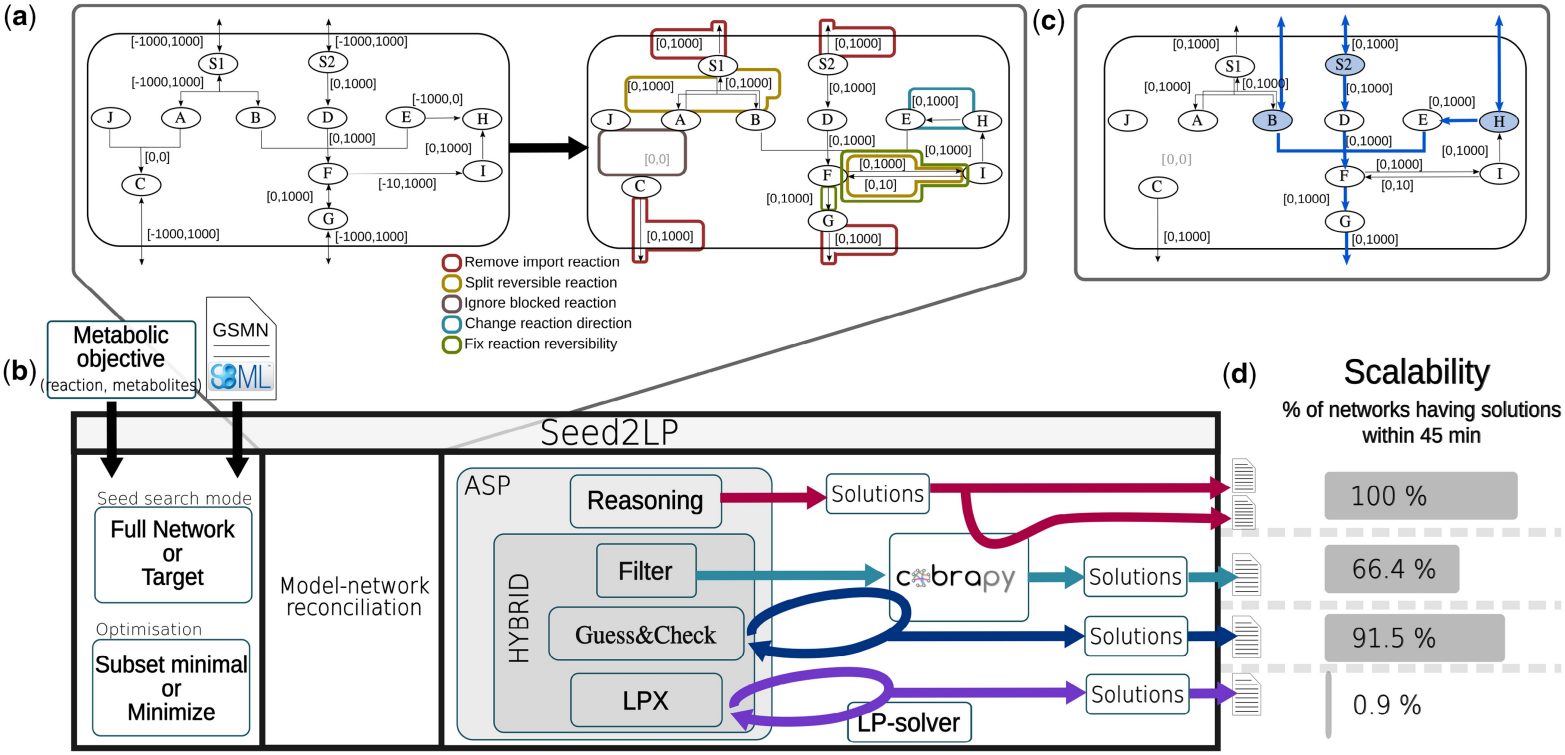
Overview of Seed2LP. (a) Network normalization transformation. The first network is the original example, and the second is the normalized network with all modifications highlighted: (i) reversible reactions are divided into two reactions, (ii) reactions with negative boundaries are reversed, (iii) import directionality of exchange reactions is blocked but export is maintained, and (iv) reactions with boundaries to 0 are deleted. (b) Illustration of Seed2LP. Inputs are a GSMN (SBML format) and a metabolic objective. The two main modes are *Full Network* (all metabolites must be reachable) or *Target* (a set of metabolites must be reached). Optimizations for the size of seed sets are subset minimal or minimize. Model-network reconciliation precedes seed inference, for which several solving modes are available. All methods use ASP; reasoning does not include FBA but it can be checked *a posteriori*. The three other methods are hybrid: *Hybrid-filter* returns the *n* first reasoning solutions validating FBA with COBRApy. *Hybrid-GC* checks flux during solving and eliminates supersets of solutions from the future ones. *Hybrid-lpx* uses a dedicated hybrid solver. (c) One seed solution for the previous GSMN obtained in *Target mode*, aiming at reaching *F*, the reactant of the objective reaction $F\overset{}{\to }G$. A solution using Reasoning inference is {B,S2,H}. During COBRApy validation, import reaction is reactivated for *S*2, sink reactions are created for *B* and *H*. A positive flux can be attained in the objective reaction in FBA. Activated reactions according to NE are highlighted in blue. Solutions obtained with Hybrid Seed2LP and alternative tools are detailed in Supplementary Section S3.3. (d) Seed2LP can be run with a time limit, highlighting the scalability of each solving mode, here on the 107 GSMNs from the BiGG database.

A first step is to split reversible reactions into two irreversible ones, thereby ensuring a consistent definition of reactants and products in the scope definition. For example, a reaction r: A↔B with $v_{min_{r}}=-10$ and $v_{max_{r}}=100$ is split into reactions $r^{\prime }$: A→B [0,100] and $r_{\mathrm{rev}}^{\prime }$: B→A [0,10]. Reactions having a negative lower bound flux and a null upper bound are considered as written backwards because they consume the metabolites set as products: e.g. A→B [−100,0] becomes B→A [0,100]. Reactions having both null upper and lower bounds are deleted. Finally, as existing exchange and sink reactions will artificially create seeds during the inference, it is possible to block the import of the corresponding metabolites: e.g. A↔∅ [−10,1000] becoming A→∅ [0,1000], or optionally to maintain a second reaction ∅→A [0,10]. Figure 1a illustrates the above transformations on a simple metabolic network.

In the following, we fix a *normalized* metabolic network N over metabolites M and reactions R and bounds $\nu_{\min}$ and $\nu_{\max}$.

#### 3.1.2 Seed search

Seeds are a subset S of the network’s metabolites M that ensure the satisfiability of a metabolic objective. Such an objective can be defined as a set of metabolites or as a reaction $T=\{(m_{\mathrm{obj}}\subseteq M)\cup (r_{\mathrm{obj}}\in R)\}$. The constraints for satisfiability of the objective vary depending on the formalism used for modelling: NE [Equation (3d)] and/or FBA [Equations (3a)–(3c)]:

(3a) X.ν=0

 

(3b) $$\nu_{r_{\mathrm{obj}}}>0\;(\text{or}\;\text{maximize}\;\nu_{r_{\mathrm{obj}}})\;$$

 

(3c) $$\nu_{\min}\leq \nu \leq \nu_{\max}$$

 

(3d) $$m_{\mathrm{obj}}\subseteq \mathrm{Scope}(N,S)$$

In practice, if metabolites are set as targets, a special case where T={M∪R} is referred to as ‘full network’ mode, leading the reachability of the entire set of metabolites to be the metabolic objective. Alternatively, while setting a metabolic reaction $r_{\mathrm{obj}}$ as an objective is straightforward in FBA, it leads to $\mathrm{reactants}(r_{\mathrm{obj}})$ to be set as targets in the NE formalism. Once the set of seeds is predicted, they are included in the GSMN by adding an exchange (respectively a sink) reaction for each seed if the corresponding metabolite is extracellular (respectively cytosolic) for FBA simulation. Seed inference can rely on the NE formalism or hybrid NE–FBA formalism. We detail below their characteristics.

##### 3.1.2.1 Reasoning-based seed inference

Reasoning-based seed inference aims at identifying sets of seeds S satisfying Equation (3d) for the normalized GSMN N under the definition of the scope in Equation (2). In the case where $T=\{M\cup R^{\prime }\}$, external metabolites of the GSMN are identified as reactants of reactions that are never produced, or solely through transport reactions, and assigned to seeds to initiate scope calculation; remaining seeds being selected from the rest of the metabolites absent from this initial scope. For other cases ($T=\{m_{\mathrm{obj}}\in M\}$), the problem is solved in ASP by describing a reverse scope problem identifying a set of putative seed candidates starting from the reactants of reactions producing targets until it reaches a fixed point: seeds are subsets of those:

(4a) find S⊆M

 

(4b) such that T⊆Scope(N,S)

Note that if S is a solution, then, any superset of it is also a solution. Therefore, we focus on enumerating *subset-minimal* solutions: sets S such that no subset is a solution.

Seed inference with NE does not ensure the satisfiability of steady-state constraints in Equation (1b), and may permit the accumulation of metabolites (Fig. 1a). Equation (1b) can be qualitatively approximated by an additional, optional, constraint on the seed search, ensuring that all metabolites in the scope have to be consumed by at least one reaction whose reactants are in the scope (*no-accumulation* mode):

(5) $$\begin{array}{c} \begin{array}{cc} \forall m\in \mathrm{Scope}(N,S), & \exists r\in R \end{array}\\ \begin{array}{c} s.t.\;\;\;m\in \mathrm{reactants}(r)\\ \text{and}\;\;\mathrm{reactants}(r)\subseteq \mathrm{Scope}(N,S) \end{array} \end{array}$$

##### 3.1.2.2 Hybrid NE–FBA seed inference

Identifying sets of seed satisfying constraints from Equation (3), thus mixing Boolean and linear constraints with subset minimization can be performed with different solving strategies. We study four approaches here:

- *Hybrid-lpx*, which employs a direct encoding of the full hybrid constraint problem, and use a dedicated solver, clingo-lpx, which intertwines logical reasoning with simplex-based checking of linear equations for propagating constraints.
- *Hybrid-filter*, which performs the reasoning-based inference and then filters the subset-minimal solutions with *a posteriori* FBA constraints checking.
- *Hybrid-GC*, which implements a so-called *guess-and-check* solving approach: a pure reasoning-based and subset-minimal solution is first identified. If it fails the linear constraints, new Boolean constraints are injected in the logic program to mark the set as inconsistent. This algorithm primarily aims at identifying sets of seeds which are subset-minimal for the NE-FBA problem, while being not minimal for the NE problem.
- *Hybrid-GC* ${}_{\mathrm{div}}$ , which extends the algorithm of *Hybrid-GC* with dynamic solver heuristics for increasing the diversity of partial enumeration of solutions. Indeed, due to their search algorithm, logic solvers tend to give successive solutions that are very similar. The heuristics we inject after each solution biases the choices made by the solver to reduce the intersection among the solutions.

##### 3.1.2.3 External validation with COBRApy

Whether from *a posteriori* seed inference with the reasoning mode or *Hybrid-filter* or during solving in *Hybrid-GC*, COBRApy (Ebrahim *et al.* 2013) solves the FBA problem and assesses whether the seed sets enable a positive flux in the biomass reaction. First, the exchange reactions are identified and the boundaries are changed to remove the existing imports if any, so that the inferred seeds will be the unique inputs to the model. For all seeds in a solution, the program will either reopen the import flux bound of its corresponding exchange reaction if any, or create a sink reaction otherwise. The flux in the objective function is then maximized and satisfiability of Equation (3) is ensured if a positive flux is obtained.

#### 3.1.3 Implementation

The above methods are implemented in a Python package called Seed2LP. The input is a GSMN in SBML format which will be normalized (see section Section 3.1) prior to seed inference. Existing exchange reactions that will act as seeds can be kept or discarded (default behaviour). Seed2LP can be run with two different seed searching modes: *Full Network* mode which ensures that all metabolites are in the scope, and the *Target* mode that focuses on a subset of metabolites. Details about the implementation are available in Supplementary Material.

##  

Further details about solutions obtained on the toy network with the distinct solving modes of Seed2LP, as well as with MILP and graph-based approaches, are available in Supplementary Material.

### 3.2 NE outperforms graph-based analysis for seed inference

Seed inference was performed on 107 curated GSMNs from the BiGG database (Norsigian et al. 2020) in order to validate the relevance of Seed2LP and results were compared to other approaches (see Supplementary Methods). Results are described below. Figure 1 illustrates the underlying concepts of seed inference with Seed2LP. NetSeed (Carr and Borenstein 2012) uses the graph structure of the GSMN (see Supplementary Methods) to identify non-produced compounds called seeds. As it works at the genome scale and does not permit inferring seeds for a subset of target metabolites, it was compared to Seed2LP in the *Full Network* setting. In order to compare the graph-based approach of NetSeed to a qualitative model of metabolic producibility (NE), we ran Seed2LP with the reasoning mode and assessed the quality of seed prediction by verifying the possibility to hold a positive flux in the biomass reaction with FBA. A maximum of 1000 seed solutions were computed for each GSMN by each tool with a 45 min timeout for Seed2LP, and with no timeout for NetSeed, which never lasted more than a few minutes for computation.

**Figure 2.**
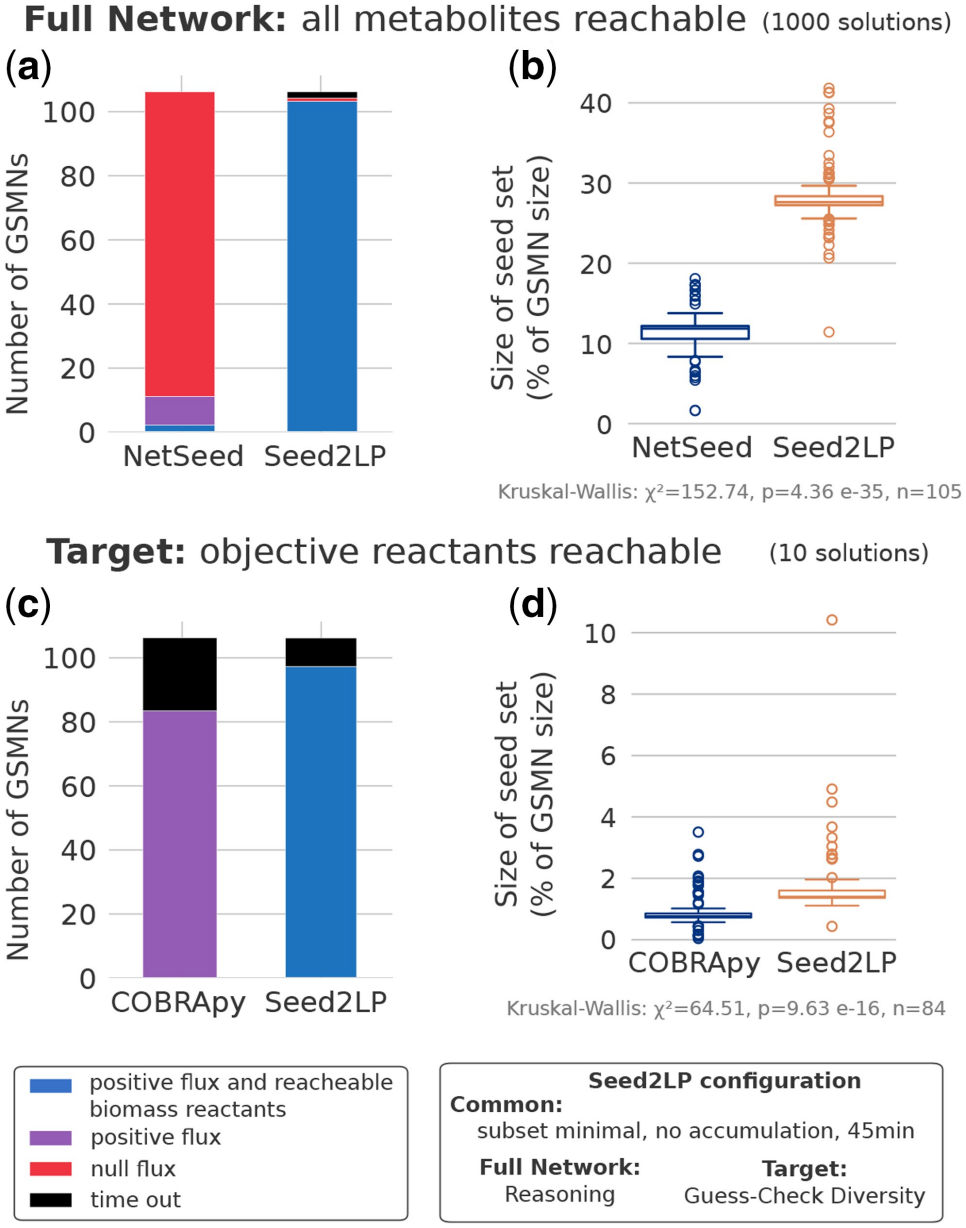
Seed inference with Seed2LP, NetSeed or COBRApy minimal_medium function. (a, b) Comparison between NetSeed and Seed2LP in *Full Network* solving mode, generating up to 1000 solutions. (c, d) Comparison between Seed2LP and COBRApy in *Target* solving mode, generating up to 10 solutions. (a, c) Number of GSMNs having all solutions validating FBA and ensuring biomass reactant reachability (blue) or all solutions validating FBA only (purple), or not validating FBA (red), or in timeout (black). (b, d) Size of seed sets described as the proportion of the total number of metabolites per GSMN. (b, d) illustrate of the average value by GSMN if several solutions were obtained. Only results from the 105 (Seed2LP versus Netseed) and 84 (Seed2LP versus COBRApy) GSMNs for which both NetSeed and Seed2LP or COBRApy and Seed2LP find solutions are plotted.

Results are depicted in Fig. 2a and b. NetSeed finds at least one solution set for all 107 GSMNs, while Seed2LP times out for 2 of them. We show in Supplementary Material that releasing the *no-accumulation* of Seed2LP’s inference results to no timeout. It must be noted that the problem solved by Seed2LP involves NE-reachability constraints, which aim at ensuring stronger properties on the inferred seeds than graph-based analysis as implemented by NetSeed. One of the consequences, besides higher computational cost, is that sets of admissible seeds are typically larger. However, as emphasized in Fig. 2a, the seeds inferred by Seed2LP show a drastic improvement for both the satisfiability of the NE-reachability property and presence of flux at steady state in all reactions. In average, 848 ± 349 and 889 ± 282 solutions could be computed by NetSeed and Seed2LP, respectively, out of the 1000 upper bound set up in the benchmark. When applying FBA to solutions computed by NetSeed and Seed2LP, we observed a consistency at the GSMN level: either all solutions for a given GSMN held a positive flux in the biomass reaction or none did. Over the 105 GSMNs instances processed with Seed2LP, 104 have seeds which validated FBA and NE constraints on biomass reactants. In contrast, only 11 GSMNs validated FBA with NetSeed solutions, among which two satisfied NE constraints (Fig. 2a). Sets of seeds computed by Seed2LP are larger than those of NetSeed (average of averages by GSMN 28.2% versus 11.5% of the total number of metabolites in the GSMN), which is expected in a context where the entirety of metabolites are expected to be NE-reached (Supplementary Fig. S3c and e). Additional results on solution sets are presented in Supplementary Material, together with a comparison between Seed2LP and the graph-based seed inference method implemented in PhyloMInt (Lam *et al.* 2020).

### 3.3 Hybrid NE–FBA is a good trade-off between scalability and numerical accuracy for seed detection

We assessed the relevance of *Reasoning* and *Hybrid NE-FBA* solving modes of Seed2LP on the same benchmark of 107 GSMNs, with a time limit of 45 min or a limit of 1000 seed solutions computed. The use case of this benchmark was to ensure the reachability of biomass reactants (reasoning and hybrid modes) and a positive flux in the biomass reaction (hybrid modes), these two objectives being more biologically relevant than reaching the whole set of metabolites.

Results are depicted in Table 1. *Hybrid-lpx* mode returned no seed solution before the timeout, indicating that this mode is not applicable to genome scale. *Hybrid-lpx* uses the hybrid ASP-simplex clingo-lpx solver: alternative hybrid modes in Seed2LP rather generate solutions with ASP and validate FBA directly during the solving (*Hybrid-GC)* or *a posteriori* (*Hybrid-filter*).

**Table 1.** Seed2LP results on 107 GSMNs (target seed searching mode, subset-minimal solutions) with several inference methods.

Solving mode	Nb. of net. with sol.	Nb. of net. with ≥1 sol. FBA	Nb of net with all sol. FBA
*Reasoning*	107	73	10
*Hybrid-filter*	71	71	71
*Hybrid-GC*	90	90	90
*Hybrid-GC* ${}_{\mathrm{div}}$	98	98	98
*Hybrid-lpx*	0	0	0

The second column indicates the number of GSMNs for which solutions are obtained. The third (resp. fourth) column indicates the numbers of GSMNs for which at least one (resp. all) solution(s) enable the biomass reaction to carry flux in FBA.

Several criteria can be used to evaluate the relevance of seed inference: obtaining one or several solutions that ensure the NE reachability of biomass reactants, ensuring a positive flux in the biomass reaction, or both of those. Results in Table 1 indicate that the *Reasoning* mode scales the best with solutions obtained for all GSMNs, and 1000 solutions for each. Hybrid modes found solutions for less GSMNs before timeout, between 66.4% (*Hybrid-filter*) and 91.6% (*Hybrid-GC*${}_{\mathrm{div}}$).

All solutions provided by the hybrid modes succeed at FBA validation, as expected by design. *Hybrid-GC* method which adds constraints to the ASP solver to exclude previous sets of seeds (and their supersets) from future solutions helps to obtain more GSMNs with solutions (+19 wrt to *Hybrid-filter*). Enhancing the diversity of solution contents with *Hybrid-GC*${}_{\mathrm{div}}$ further increases the number of GSMNs with solutions (+8 wrt to *Hybrid-GC*).

The reasoning mode does not ensure FBA validation: only 9.3% of GSMNs exhibited a positive flux in all of their solutions. However, for 68.2%, at least one generated solution had a positive flux, highlighting that this mode is not only scalable but can also provide FBA-compliant solutions. Identifying the latter requires an *a posteriori* validation with COBRApy though, which is implemented in Seed2LP. Including this validation in the solving, as performed by the *Hybrid-filter* increases the computational time, which explains the smaller number of GSMNs with a solution for the latter mode. We detail in Supplementary Material the computational time needed for the computation of a single solution in all four inference modes.

Above results were obtained with a subset-minimal optimization. It is possible to use a minimize optimization in Seed2LP but this setting led to less GSMNs having a solution, and less solutions by GSMN in 45 min. In this setting, *Hybrid-lpx* provided two solutions for the core network of *Escherichia coli* (95 reactions and 72 metabolites) using the minimize solving mode. Finally, we compare in Supplementary Material seed inference results obtained with Seed2LP or with an alternative flavour of the NE algorithm, ensuring self-regeneration of cycles in GSMNs. The latter approach provides seed sets of smaller size than Seed2LP but less solutions ensure a positive flux in the biomass reaction.

We explored the ability of an existing MILP implementation to propose seed sets satisfying FBA and NE constraints with the function minimal_medium implemented in COBRApy (Ebrahim *et al.* 2013) that minimizes components of a defined medium while ensuring a positive flux in the objective reaction. While this implementation better suits curated GSMNs for which an existing medium is available, we could design comparable use-cases for Seed2LP and COBRApy enabling a search among all metabolites, including internal ones (see details in Supplementary Material). We generated up to 10 solutions for each of the 107 BiGG GSMNs with a 45-min timeout. COBRApy was compared to Seed2LP in the *Target* setting using *Hybrid-GC*${}_{\mathrm{div}}$  *solving mode*.

Results are depicted in Fig. 2c and d. COBRApy finds up to 10 solutions for 84 GSMNs, while Seed2LP finds solution sets for 98 GSMNs. By design, both tool satisfy FBA constraints. Biomass reactants reachability is not a constraint for COBRApy, and none of the results comply with NE. Sets of seeds computed by Seed2LP are slightly larger than those of COBRApy in average (1.79% versus 0.92% of the total number of metabolites in the GSMN, P<0.001). We detail further the results and computation times in Supplementary Material, showing that Seed2LP is overall better suited to explore efficiently the solution space.

### 3.4 Exploration of seed diversity in solutions

We analyzed further the content of seeds inferred by Seed2LP and the differences across solving strategies by focusing on the GSMN of *Acinetobacter baumannii* AYE (iCN718) (888 metabolites, 1015 reactions). Again, the metabolic objective was the production of biomass precursors (*Reasoning* and *Hybrid* modes) in addition to a positive flux in the biomass reaction (*Hybrid* modes). In the benchmark results presented in Table 1 (45 min or 1000 solutions), the *Reasoning* mode raised 1000 solutions (465 FBA-validated), whereas *Hybrid-filter* (H${}_{F}$), *Hybrid-GC* (H${}_{GC}$) and *Hybrid-GC*${}_{\mathrm{div}}$ (H${}_{\mathrm{GCdiv}}$) only generated 149, 116 and 111, respectively, before the timeout. While the sizes of seed solutions are relatively small (between 9 and 27 metabolites), enumerating solutions enables diverse metabolites to be selected as seeds, which can be biologically relevant for experimental validation. As such, the union of metabolites occurring in the solutions described above was of size 161, 67, 98 and 118 for *Reasoning*, H${}_{F}$, H${}_{GC}$ and H${}_{\mathrm{GCdiv}}$, respectively, highlighting that the latter mode indeed reaches more diverse solutions than the other hybrid modes.

**Figure 3.**
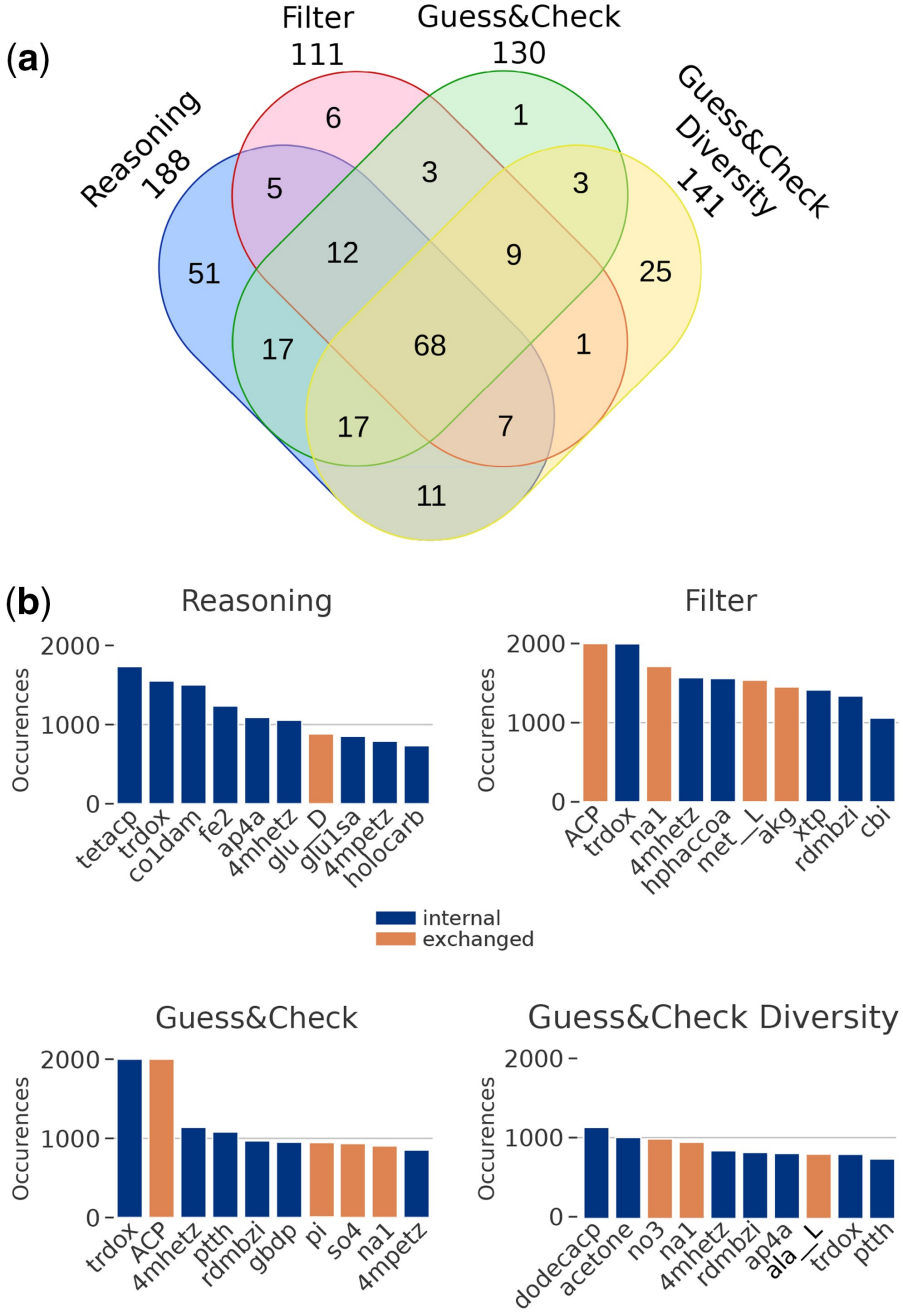
Seed inference in the iCN718 GSMN. (a) Venn diagram illustrating the contents of solution unions in the four Seed2LP inference modes in 2000 solutions. (b) Barplots depicting the 10 most frequent metabolites in seed solutions from the four modes. Orange metabolites correspond to molecules originally imported in the BiGG model, and blue metabolites are internal. In both subfigures, seed unions were de-replicated before analysis, i.e. the same molecule in two different compartments was considered identical.

In order to survey the diversity of seed solutions captured with larger enumerations, we ran Seed2LP until reaching 2000 solutions. It is worth noting that while the *Reasoning* mode computed this number of solutions in 6 seconds on a laptop, it took 8 days on a computing cluster for the *Hybrid-GC* mode to do so. However, only 381 solutions validated FBA among the *Reasoning* ones. Sizes of the union of solutions show a low increase compared to the 45 min or 1000 solutions benchmark (199, 117, 135 and 149 for Reasoning, H${}_{F}$, H${}_{GC}$ and H${}_{\mathrm{GCdiv}}$, respectively), indicating that while the problem of seed inference has a high combinatorics, the number of metabolites involved in solutions is relatively small and does not increase much in larger enumerations. The relevance of *Hybrid-GC*${}_{\mathrm{div}}$ over the other hybrid modes is observable in both the 45-min and 2000-solution benchmarks: after the *Reasoning* mode, it is the one that raises the largest union of solutions and therefore explores the most the solution space (Fig. 3a, Supplementary Material).

We finally surveyed the most frequent seeds across all solutions and the four modes, distinguishing internal metabolites from those which were imported in the original model (*n* = 120 metabolites imported through exchange or sink reactions). Figure 3b depicts the 10 most frequent metabolites per mode. Only one exchanged metabolite occurs among the most frequent one in reasoning mode, whereas three or four appear in the hybrid modes. We can argue that the solutions validating FBA propose more exchanged metabolites in the solutions than *Reasoning*. The emphasis on diversity in the *Hybrid-GC* and especially *Hybrid-GC*${}_{\mathrm{div}}$ modes is visible as few of the most frequent metabolites occur in more than a thousand solutions (see also Supplementary Figs S9 and S10). It is worth noting that the medium encoded in the original GSMN enables reaching 31% of metabolites with NE, and no biomass reactant, an observation that can be explained by the steady-state modelling of FBA, in opposition to the more stringent initial-state modelling of NE (Frioux *et al.* 2019).

## 4 Discussion

We developed a seed detection tool, Seed2LP, relying on a Boolean approximation model of metabolic producibility, that can be combined with FBA to infer growth medium nutrients. Obtaining chemically defined or synthetic media has multiple applications in microbiology, especially for fastidious and uncultured microorganisms (Stewart 2012). Microbial dark matter, identified with techniques such as metagenomics impedes the understanding of most ecosystems harbouring a wide diversity of organisms (Mu *et al.* 2021, Kapinusova *et al.* 2023), but other use cases exist, for instance in the context of endosymbiont bacteria that cannot be grown outside of their hosts (Masson and Lemaitre 2020). Solving the culturability bottleneck has multiple applications, given the wide range of metabolic functions with biotechnological interest that microorganisms can provide (Lewis *et al.* 2021, Rämä and Quandt 2021). Systems biology and reverse ecology, through the implementation of dedicated computational models, can help addressing these objectives by suggesting necessary nutrients for growth.

Seed2LP is highly flexible, allowing users to choose between different seed search methods, focusing solely on the producibility of reactants of an objective reaction, or on the reachability of all metabolites in the network. Multiple parameters enable constraining seed inference, and solving modes based on *Reasoning* or *Hybrid NE–FBA* customize further the search. The reasoning NE-based model is highly scalable and efficient, often providing a thousand solutions well before the 45 min timeout of our benchmark, all ensuring that reactants of the objective reaction are reachable. We chose the ability of models to carry flux in FBA as a validation method, an approach that is widely acknowledged in systems biology. We showed that Seed2LP *Reasoning* outperforms other existing methods such as graph methods (NetSeed) or alternative NE flavours (Supplementary Material). Even if constraint-based modelling such as FBA is independent of NE, we observe that for the majority of GSMNs in both *Target* and *Full Network* modes, at least one solution ensures a positive flux in the biomass reaction. Hybridizing NE with FBA ensures biomass production but comes with a computational cost that results in fewer solutions being generated, and a smaller solution space being explored. We demonstrate nonetheless that a first solution can be obtained within a few seconds for most GSMNs. *Reasoning* and *Hybrid* modes achieve varying level of diversity during seed enumeration, and among the Hybrid ones, *Hybrid-GC*${}_{\mathrm{div}}$ reaches the most diverse solution contents. Comparing Seed2LP *Hybrid* to an MILP implementation ensuring FBA highlighted the computational efficiency of the former in exploring the solution space, and the relevance of subset minimality optimization.

The topology of GSMNs is very diverse and can facilitate or complicate the inference of seeds, resulting in solving modes that can be more or less efficient depending on the network. As such, there is no optimal combination of parameters suitable for any GSMN, and one may benefit from testing and comparing several of them. While the inference can be fully automatized and performed systematically on a large collection of GSMNs, Seed2LP’s flexibility aims at making it useful in practice for experimental applications. It can be used in a back-and-forth process with bench work to partly automatize metabolic modelling-based medium inference, a use case that typically requires curation (Tejera *et al.* 2020, Nev *et al.* 2024). Selecting seeds among a subset of metabolites or forbidding some molecules to be used as seeds may prove useful in such iterative process. Seed2LP is a support to find the nutrient-associated missing link among the numerous environmental factors impacting culturability: nutrients, pH, osmotic conditions, temperature and others (Stewart 2012). A limitation of Seed2LP is therefore that it only considers metabolism to predict growth. Impact of molecule concentration, regulation processes or the dynamics of metabolism are additional facets whose analysis could be carried with more complex computational models, for instance in between seed inference and experimentation.

From a computational standpoint, our experiments suggest that the pure Boolean constraints of NE together with non-accumulation constraints form an efficient abstraction of the complete *Hybrid NE–FBA* problem, with a limited false positive rate. This tends to justify the use of pure logic-based solving methods to pre-process the solution space rather than rely on hybrid solving techniques, being either logic programming combined with LP or MILP. While we show here that both NE and FBA are compatible, it has to be noted that cofactors or currency metabolites identification may be of importance for NE specifically (Belcour *et al.* 2020), and more generally for reasoning-based or graph-based approaches. Seed2LP includes the possibility to forbid or force certain seed compounds, which can be used to consider those metabolites accordingly. Future work may investigate further discrete constraints that would make the Boolean over-approximation even more accurate, reducing the need for linear constraints checking, and alternative implementations using MILP-based technologies.

While use cases presented in this paper concern individual populations, the question of seed inference can be extended to co-culture contexts (Stewart 2012), where interactions among species and division of labour can impact the minimal requirements of the community (Lewis *et al.* 2021). Seed inference in that case could answer several objectives, ranging from stabilizing or guiding the community to designing medium by taking cooperation into account or favouring it.

## Supplementary Material

btaf140_Supplementary_Data

## Data Availability

Seed2LP is available on https://github.com/bioasp/seed2lp. Datasets, raw results and analysis scripts are available on https://doi.org/10.57745/OS1JND for reproducibility purposes.
