# Supplementary material for: Seed2LP: seed inference in metabolic networks for reverse ecology applications
Source: Bioinformatics. 2025 Mar 31;41(4):btaf140. doi: 10.1093/bioinformatics/btaf140 (PMC12007882; doi:10.1093/bioinformatics/btaf140)
Supplement: btaf140_Supplementary_Data [file btaf140_supplementary_data.pdf]

Supplementary Content for *Seed2LP: seed inference in metabolic networks for reverse ecology applications*

Chabname Ghassemi Nedjad, Mathieu Bolteau, Lucas Bourneuf, Loïc Paulevé and Clémence Frioux

1. A flexible tool for seed inference in metabolic networks - Details about the implementation

Seed2LP is a Python package that infers seed metabolites, i.e., input nutrients, that would enable a metabolic objective to be sustained in a metabolic network according to a simulation paradigm. The core of Seed2LP functioning is machine-reasoning with ASP, and a simplification of metabolic reachability with the network expansion (NE) algorithm. Additionally, it implements various *Hybrid FBA-NE* solving modes in order to ensure the resulting models with the inferred seeds satisfy FBA constraints (Fig. 1).

Seed2LP's *Target* and *Full Network* modes can be run with five different seed inference methods: *Reasoning*, *Hybrid-lpx*, *Hybrid-filter*, *Hybrid-GC* or *Hybrid-GC*$_{div}$ (See section on hybrid inference in Contributed Method). An objective reaction has to be provided for all modes involving the NE-FBA formalisms. If not set otherwise, default metabolic targets will be the reactants of the objective reaction described in the SBML input file. Main Figure 1 illustrates the impact of reconciling the network structure and its associated mathematical model in the SBML input, highlights the flexibility of seed search and solving modes of Seed2LP, and illustrates one solution on a toy example.

Additional constraints for seed inference can be provided to the tool: a set of forbidden seeds that can never occur in solutions, a set of existing seeds that will be completed, or a set of possible seeds among which the search will be performed. In the default behaviour, targets are excluded from the seeds to prevent trivial solutions.

Seed2LP provides (subset-)minimal size solution seeds in a json file. It implements post-validation FBA, which is particularly useful for reasoning-based seed inference that does not guarantee steady state. This validation can be used as a standalone step and is complemented by the possibility to assess the size of the NE scope from a given set of seed using MeneTools [1, 2], thereby verifying that Eq. 3b holds.

2. Material and methods

2.1. Benchmark Data

Curated GSMNs were retrieved from BiGG Database [3], using the API v2 last updated on 2019-10-31. The collection includes 108 GSMNs, but iAT_PLT_636 was discarded from the analyses because of a lack of biomass reaction. GSMNs account mostly for bacteria, but also a few eukaryotes, including the human. All GSMNs have a set of exchange reactions that we can associate to seeds. 105/107 also hold a positive flux in FBA when optimising their biomass reaction, the two exceptions being Recon1 and iCN900.

2.2. NetSeed

Seed inference relying on strongly connected components (SCC) was introduced by [4]. Briefly, SCCs are computed on the simple directed graph associated to the GSMN, these SCCs are nodes of a directed acyclic graph and the ones without input edges are the sources among which seeds are selected. This approach is implemented in NetSeed [5]. NetSeed provides the seeds associated with a GSMN, and distinguishes those that originate from the same source component by attributing them a confidence score $1/n$, n being the number of metabolites in the component. Therefore, by computing the Cartesian product of all source component, it is possible to enumerate several seed solutions. As an example, let us imagine that 2 SCC with no input are outputed by NetSeed. In the first, only one compound A. In the second, there are 3 compounds, $\{B, C, D\}$ that are listed by NetSeed with a probability of 0.33 (1/ SCC size). This will lead to 3 solutions here: $\{A, B\}$, $\{A, C\}$, $\{A, D\}$.

We downloaded the NetSeed-Perl tool from the NetSeed Website[1] on 2022-01-19 [5]. GSMNs were normalised with Seed2LP (keeping the import reactions defined in the source file and other parameters by default) prior NetSeed execution in order to ensure consistency between the network structure and associated mathematical model. NetSeed was run with default parameters. It outputs a set of metabolites corresponding to the intersection of all solutions, and a collection of metabolite sets (SCCs without inputs) among which one seed must be selected. We performed a scalar product of the sets composing seeds to get the first thousand solutions.

2.3. Analyses with PhyloMInt

The implementation of PhyloMInt seed search and graph construction was extracted from the source code version (v 0.1.0) [6] and added into a script. The original graph construction does not take into account reversible reaction and for complementary analyses such reactions were added into the graph before seed searching.

Seed inference was applied to the GSMNs normalised by Seed2LP, keeping import reaction defined in the source file, and FBA was performed on the model with the resulting union of seeds.

2.4. Analyses with COBRApy

COBRApy (v. 0.26.0) [7] provides a function `minimal_medium` minimising components of a defined medium while ensuring a positive flux in the objective reaction. The defined medium consists in a collection of exchange reactions. The function solves a MILP problem minimising the number of components in the medium, i.e. the number of exchange reactions to be kept. This implementation does not take into account the reachability of target metabolites according to the NE formalism, nor can it select compounds for which no exchange is defined, such as internal metabolites. It therefore better suits curated metabolic networks.

[1] `http://borensteinlab.com/software_NetSeed.html`

In order to compare the inference of minimal medium implemented in COBRApy to Seed2LP, we created an exchange reaction and its corresponding transport for every metabolite of the BiGG GSMNs except the reactants of the biomass, thereby building a similar search space for both tools. This also reproduces a similar behaviour to Seed2LP which forbids the selection of seeds among targets (i.e. reactants of the objective reaction) by default.

COBRApy was then run on the GSMNs with a timeout of 45 minutes for the inference of 1, then 10 set of seeds solutions.

2.5. Analyses with Seed2LP

Seed2LP is a Python (v. 3.10) package, and that uses as main dependencies: Clyngor (v. 0.3.18) and Clingo-lpx (v. 1.3.0) for managing ASP, COBRApy (v. 0.26.0) [7], MeneTools (v. 3.4.0) [2] and Metage2Metabo (v. 1.5.4) [1].

Seed2LP was run with the 107 BiGG GSMNs, using the *full-network* and *target* objective modes, reasoning and hybrid inference modes. Unless stated otherwise, the subset-minimal optimisation was used and the reasoning modes used the *no-accumulation* setting. Unless stated otherwise, the execution of the programme was stopped when 1000 solutions were obtained, or after 45 min. An additional benchmark was performed with a 45-minute timeout to assess the time necessary to obtain one solution, with 1 core and 10Gb memory.

Validation of a positive flux in the biomass reaction was performed with COBRApy within Seed2LP for both Seed2LP and NetSeed solutions. The NE scope was computed through the Seed2LP *scope* functionality, using the SBML corresponding to the corrected GSMN. The presence of all GSMN metabolites (i.e., those occurring in reactions) in the scope was verified.

An additional benchmark was performed with a 45-minute timeout in *Hybrid-*GC_{div} mode in order to obtain up to 10 solutions and compare results with COBRApy's minimising component solutions.

GSMN iCN718 was selected for further analysis and Seed2LP was run until 2000 solutions were obtained in *reasoning*, *Hybrid-filter*/*Hybrid-GC*/*Hybrid-*GC_{div} modes.

Calculation of exchanged metabolites coverage in inferred seeds consists in divising the number of exchanged metabolites (from exchange or sink reactions that import the molecule) among seeds by the total number of exchanged metabolites.

2.6. Statistics and visualisation

All plots were generated with seaborn (v. 0.13.2) [8, 9], using Python scripts to extract data, pandas (v. 2.2.2) [10] to aggregate data and manipulate tables and SciPy (v. 1.14.0) [11] for statistical tests. Unless stated otherwise, the latter were performed with the Kruskal-Wallis test [12]. In all boxplots, the box indicates the quartiles of the dataset while the whiskers extend to the rest of the distribution, except for the points determined as outliers. The Venn diagramm was computed with `http://bioinformatics.psb.ugent.be/webtools/Venn/` after retrieving the ID's of the union of metabolites for each method stripped from their prefix and compartment suffix.

3. Comparison of seed inference frameworks on a toy example

Main Figure 1 depicts the concepts of GSMN normalisation prior seed inference and the solving framework of Seed2LP on a toy example. We reproduce the toy metabolic network of Figure 1 in Figure S1 below and describe the sets of seeds predicted by Seed2LP, COBRApy's `minimal_medium` function and NetSeed on such data. In this example, the objective reaction in FBA is $F \rightarrow G$, leading F to be the target metabolite in NE.

3.1. MILP-based minimisation of medium components with COBRApy

Prior to using COBRApy's `minimal_medium` function, exchange reactions (and associated transport reactions if relevant) are created for all metabolites of the network in order to simulate a search space that is comparable with the other tools. Solving the MILP problem provides a unique solution for the network, consisting in the I metabolite only. It guarantees a positive flux in FBA and the reachability of the F target (Fig. S1a).

3.2. Graph-based seed inference relying on strongly connected components with NetSeed

NetSeed computes strongly connected components (SCC) on the simple graph of the GSMN, meaning that a reaction $A + B \rightarrow C + D$ will lead to four reactions: $A \rightarrow C$, $A \rightarrow D$, $B \rightarrow C$ and $B \rightarrow D$. SCC are groups of vertices in a graph that can each be reached from any other vertex of the SCC. The simple graph of the network from Figure S1 contains two SCC: $\{A, B, S1\}$ and $\{E, F, H, I\}$. A directed acyclic graph can be constructed from the SCC, and any SCC without any incoming edge is a source that contains a seed. If the source contains a unique vertex, it is a seed with a probability of 1; if it is a SCC of size n, a seed has to be selected from the SCC and will have a probability $1/n$.

In the toy (Fig. S1b), two sources are inferred: $S2$ and the SCC containing $\{A, B, S1\}$. The Cartesian product of these sets leads to three solutions of two seeds each, containing $S2$ and one seed among (A or $S1$ or B).

None of these solutions enables to reach the reactants of the biomass reaction with NE, nor to obtain a positive flux in the objective reaction $F \rightarrow G$.

3.3. Reasoning-based and hybrid FBA-NE seed inference with Seed2LP

Seed2LP provides subset-minimal solutions satisfying NE constraints in *Reasoning* solving mode, and both NE and FBA constraints in GC_{div} mode. We detail below the results obtained with each mode on the toy example presented in main Fig. 1. The objective function for FBA and Seed2LP hybrid modes is the reaction $F \rightarrow G$.

Inference in *Reasoning* mode provides 9 solutions, among which one is of size 1: I, similar to COBRApy's minimal-size solution, and 8 are of size 3 and can be summarised in the following formula: $(B \vee S1) \wedge (H \vee E) \wedge (S2 \vee D)$. I cannot be selected instead of H or E in size-three solutions because it is already part of a smaller (size 1) solution. By design, all 9 solutions satisfy NE constraints. Seed2LP enables filtering

Reasoning solutions for those satisfying FBA constraint with the *Filter* solving mode: 5 solutions are kept out the 9 ones, discarding solutions with $S1$ that lead to an accumulation of A.

The *no-accumulation* option of Seed2LP prevents the selection of seeds which reach metabolites that are not consumed. Preventing accumulation to occur generates 12 solutions of size 3 each. I alone is no longer a solution because it will make E reachable but not consumed. The 12 solutions can be summarised in the following formula: $(B \vee S1) \wedge (H \vee E \vee I) \wedge (S2 \vee D)$. By design, all 12 solutions satisfy NE constraints. Filtering for solutions ensuring steady-state compliance selects 8 solutions summarised by the following: $(S1 \wedge I \wedge (S2 \vee D)) \bigvee (B \wedge (E \vee H \vee I) \wedge (S2 \vee D)$ (identical to GC_{div} mode without accumulation below).

Finally, the hybrid solving mode GC_{div} provides 5 solutions, among which one of size 1 I, and the remaining of size 3, summarised in: $B \wedge (H \vee E) \wedge (D \vee S2)$. The *no-accumulation* option of GC_{div} generates 8 solutions summarised by the following: $(S1 \wedge I \wedge (S2 \vee D)) \bigvee (B \wedge (E \vee H \vee I) \wedge (S2 \vee D)$ (Fig. S1c). Those solutions hold both NE and FBA constraints. The flux in FBA circulates either through I the minimal-size solution provided by COBRApy, or through the combination of B and two other metabolites. $S1$ cannot be used in FBA because it would lead to an accumulation of A. The remaining metabolites in the solutions are necessary for the NE constraint.

Changing the reversibility of the reaction $F \leftrightarrow I$ to $F \rightarrow I$ in the toy example, results in {B,D} being proposed as a minimal medium by COBRApy MILP implementation. This solutions ensure flux into the Biomass $F \rightarrow G$ but the reachability of the metabolite F is not ensured using Network Expansion (metabolite E or I or H is needed to reach F in NE). More details in Section 8 Seed inference with COBRApy of this document.

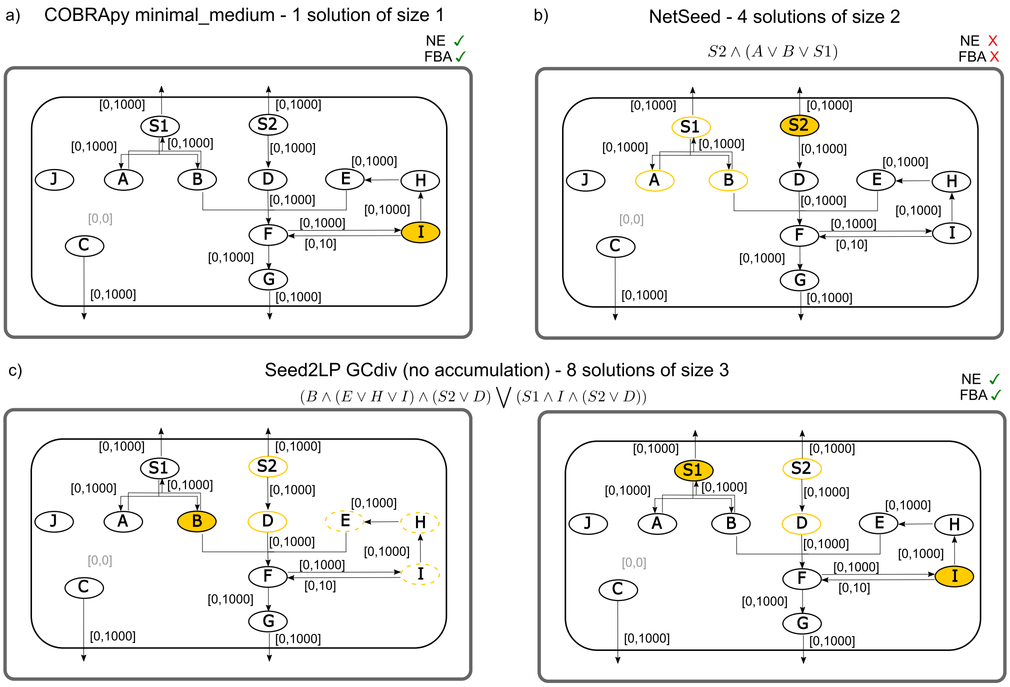

Figure S1: Toy network for illustration of the seed inference approaches. (a) COBRApy provides a unique solution. (b) NetSeed provides 4 solutions, each consisting of 2 seeds. (c) Seed2LP's hybrid solving mode GC_{div} with the *no-accumulation option generates 8 solutions of 3 seeds, summarised in the two figures: 6 solutions in the left one, 2 in the right one. In (b), (c), nodes with similar outline are alternative seeds in the solutions. For instance in (b), a solution consists in* $S2$ *and either* A*,* B*, or* $S1$.

4. Computational time for the inference of the first solution according to Seed2LP solving modes

Figure S2 illustrates the discrepancies in computation time across the four solving modes and in *Full Network* or *Target* search modes. Results indicate that *Hybrid-filter* takes the most time to retrieve solutions, especially in *Target* search mode.

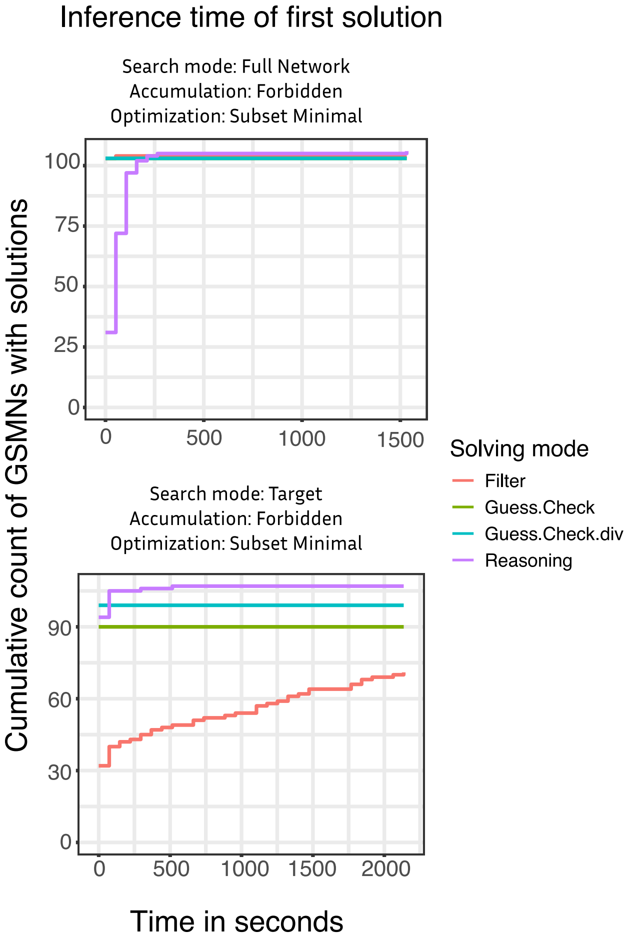

Figure S2: Solving time for the inference of a single seed solution in *reasoning*, *Hybrid-lpx*, *Hybrid-filter*, *Hybrid-GC* or *Hybrid-GC_{div}* modes. The cumulated number of GSMNs with a solution over time and before a 45 minute timout is illustrated. *Full Network* and *Target* modes are tested.

5. Subset Minimal versus minimal-size optimisations

Finding minimal set of solutions is more computationally expensive than finding subset minimal solutions. We compared the two optimisations using all seed searching modes (*Full Network* or Target) and all seed inference methods (*Reasoning*, *Hybrid-lpx*, *Hybrid-filter*, *Hybrid-GC* or *Hybrid-GC_{div}*). We analysed the sizes of seed solutions with respect to the size of the GSMN, and the coverage of exchange metabolites by sets of seeds. As the minimise optimisation does not raise solutions for all GSMNs, we restricted our analyses to GSMNs for which both optimisations gave results. We averaged all solution sizes per GSMN and compared the distribution of these values for the two optimisation modes with Kruskal-Wallis tests.

The proportion of exchange metabolites covered by seeds does not significantly change in subset-minimal versus minimal solutions regardless of the seed searching modes and inference method used. No significant difference occurs in the size of seed solutions in *Full Network* mode. In *Target* mode, only reasoning inference provides significantly smaller sets of seeds ($n = 12$, $\chi^2 = 4.81$, $p = 0.028$).

Overall, subset-minimal and minimal optimisations provide very similar results. The added-value in

scalability associated with the subset minimal mode justifies its default usage in Seed2LP.

6. Seed inference with NetSeed

Results of the comparison between Seed2LP, in *Reasoning* solving mode and *full network* search mode, and Netseed are illustrated in Figure S3. We provide a description of subpanels a) and c) in the main text.

We studied the coverage of exchange metabolites, i.e., the medium used for simulation as described in the original BiGG GSMNs, by seeds (Fig. S3b). Seed2LP includes a higher proportion of such metabolites than NetSeed (average of averages by GSMN of 89.4% vs 30.6% respectively). We then surveyed the ability for seeds computed by NetSeed to reach metabolites of the GSMN or biomass precursors using the NE algorithm. By design, all metabolites, therefore including the biomass reactants, are reachable from the seeds computed by Seed2LP. Only 69.4% and 68.6% in average of biomass reactants and of all metabolites respectively were reachable from the seeds proposed by NetSeed (Fig. S3d, e). This indicates that while NetSeed infers seeds from the global GSMN, the purely graph-based solving, and its associated simplifications (e.g. GSMN transformed into a simple graph: a reaction $A + B \to C$ is transformed into two reactions $A \to C$ and $B \to C$) do not enable the reachability of all nodes. On the contrary, the modelling approach of NE as used in Seed2LP ensures this reachability and, despite being qualitative as well, is more consistent with FBA numerical constraints.

We additionally tested the impact of the network normalisation step on NetSeed, performed as pre-treatment by Seed2LP. If no normalisation step is performed, the same number of GSMNs passes FBA validation. Likewise, the number of solutions enabling to reach (network expansion) biomass reactants decreases when normalising and removing all import reactions prior inference, while the number of GSMNs having a positive flux in the biomass reaction remain constant.

Lastly, we assessed the effect of the absence of accumulation in NE for reasoning-based seed inference with Seed2LP. Removing this constraint enabled to obtain seeds for all 107 GSMNs within the time limit, among which 106 harboured FBA-compliant solutions.

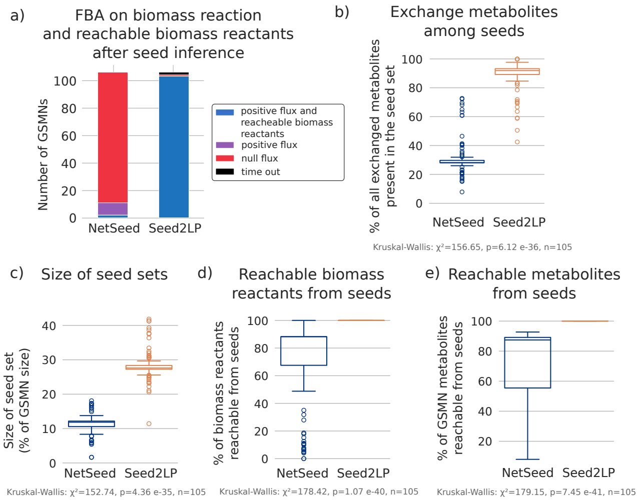

Figure S3: Seed inference with Seed2LP and NetSeed. a) Number of networks having all solutions validating FBA and ensuring biomass reactant reachability (blue) or all solutions validating FBA only (purple) or not (red), or in timeout (black). b) Proportion of all original exchange metabolites among the inferred seeds. c) Size of seed sets described as the proportion of the total number of metabolites per GSMN. d) Proportion (%) of reachable biomass reactants from the seeds out of the complete set of biomass reactants. e) Percentage of all GSMN metabolites reachable from the seeds. b), c), d), e): Distribution of the average proportions computed by GSMN if several solutions were obtained for a given GSMN. Only results from the 105 GSMNs for which both NetSeed and Seed2LP find solutions are plotted.

7. Seed inference with PhyloMInt

The underlying graph-based inference of seed as implemented in NetSeed was reused in several tools, notably for the purpose of inferring pairwise microbial interactions from GSMNs by comparing their respective sets of seed metabolites [13, 14]. More recently, PhyloMInt [6] implemented a similar approach of seed inference as a prerequisite for quantifying metabolic interactions between microbial species. While seed inference is not an objective of this tool, the implementation is available in its source code. We therefore tested it as a more recently published graph-based alternative than NetSeed.

PhyloMInt uses NetworkX [15] for the computation of SCC. NetworkX implements Tarjan's algorithm [16] with Nuutila's modifications [17] to define strongly connected components. While the approach is similar to NetSeed, the PhyloMInt implementation only provides one solution that does not enable identifying alternative seeds originating from the same source component. A confidence value is nonetheless associated with each seed. We applied the PhyloMInt implementation of seed detection to the normalised GSMNs of the BiGG database.

We compared the set of seeds in the unique solution to the prediction of Seed2LP in *Reasoning* solving mode and *full network* search mode. For each inference of seeds, FBA was applied to verify the flux in the biomass reaction, and reachability of the biomass reactants, and of the entire set of GSMN metabolites was assessed.

Results are illustrated in Figure S4. A first comment is that a unique solution is returned by PhyloMInt by design (union of all seeds) whereas Seed2LP produces up to 1000 solutions in the 45 minute timeout. Seed2LP obtains seed sets consistent with FBA and NE (biomass reactants) for 104 GSMNs while PhyloMInt

has solutions validating FBA constraint for 88 GSMNs, with 6 GSMNs also ensuring biomass reactants reachability. Despite PhyloMInt proposing the union of all solutions, the size of seed sets is larger for Seed2LP. The proportion of exchanged metabolites included in solutions is overall similar across tools.

Importantly, we observed that PhyloMInt's results differ from NetSeed's with much larger sets of seeds considering the union for both, which was surprising because the former presented its approach as similar to the latter's. We noticed that the difference between both resides in PhyloMInt's implementation not considering reaction's reversibility. Taking into account the reversibility of reactions in the construction of the graph prior SCC computation led 106/107 GSMNs to exhibit a union of seeds identical to NetSeed.

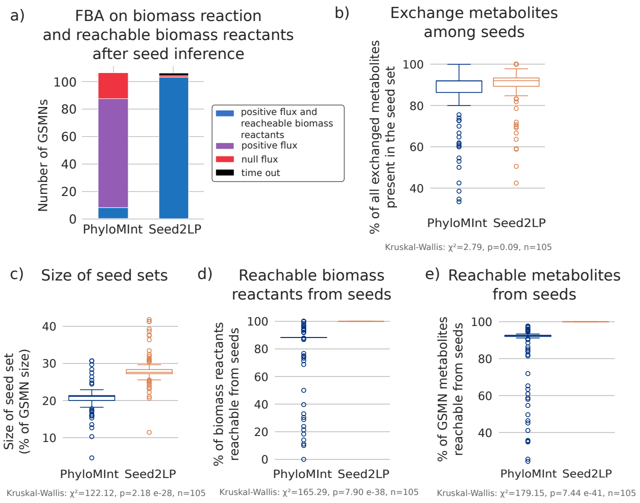

Figure S4: Seed inference with Seed2LP and PhyloMInt. The code calculating seeds in PhyloMInt was extracted and used for this experiment. A single solution consisting in the union of all seed metabolites is outputted by PhyloMInt. a) Number of networks having all solutions validating FBA and ensuring biomass reactant reachability (blue) or all solutions validating FBA only (purple) or not (red) or in timeout (black). b) Proportion of all original exchange metabolites among the inferred seeds. c) Size of seed sets described as the proportion of the total number of metabolites per GSMN. d) Proportion (%) of reachable biomass reactants from the seeds out of the complete set of biomass reactants. e) Percentage of all GSMN metabolites reachable from the seeds. b), c), d), e): Distribution of the average proportions computed by GSMN if several solutions were obtained for a given GSMN; only results from the 105 GSMNs for which both PhyloMInt and Seed2LP find solutions are plotted.

8. Seed inference with COBRApy

We used COBRApy's `minimal_medium` to compare a MILP implementation of seed inference with the approach proposed in Seed2LP (see section 2.4 Analyses with COBRApy. Results were compared with the *Hybrid-GC*$_{div}$ mode of Seed2LP (*target* inference mode). By default, both methods guarantee a positive flux in the objective reaction but only Seed2LP guarantees the NE reachability of the biomass reactants. NE-reachability was therefore assessed for all GSMNs from the predicted seeds. Because the case where a positive flux is obtained in the objective reaction in steady-state conditions despite biomass reactants not being reached in NE may not be intuitive, we provide an example in Figure S5. This example is altered from the toy GSMN of main Figure 1 and illustrates a solution of minimal medium proposed by COBRApy (enabling of compounds as potential seeds) that does not ensure the NE reachability of the reactant F of the objective reaction $F \rightarrow G$.

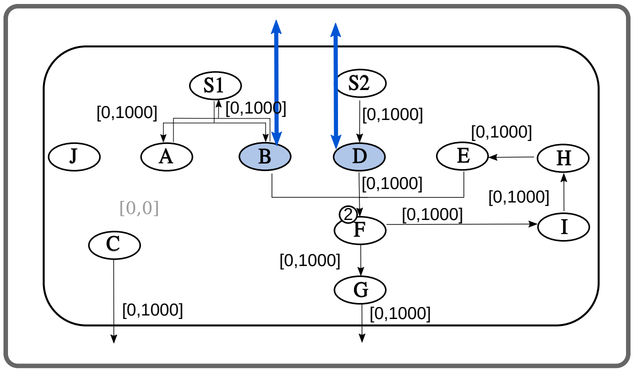

Figure S5: Adaptation of the toy example of main Fig 1. Compared to Figure 1, the reverse direction of reaction $F \to I$ has been deleted, and the reaction consuming B, D, and E now produces 2 molecules of F instead of 1. COBRApy's MILP implementation of medium inference applied to any metabolite of the network proposes $\{B, D\}$ as a set of seeds. Those seeds, despite ensuring a positive flux in the objective reaction $F \to G$, does not permit the NE reachability of F, the reactant of the objective reaction.

Figure S6 illustrates the computational time for the inference of 1 and 10 solutions. While Seed2LP finds solution for 98 GSMNs only out of 107 before timeout, it does very rapidly either for 1 or 10 solutions. The MILP implementation of the problem takes more time for the inference of a single solution but succeed nonetheless for 106 GSMNs before timeout. One network (RECON1) could not be solved due to numerical instability arising from its large size. Inferring 10 solutions takes more time and COBRApy solves less GSMNs than Seed2LP in this experiment (84 vs 98 respectively). This suggests that while MILP implementations can efficiently propose a single solution, they are less fitted to the exploration of the solution space. This is nonetheless expected because we considered a huge search space where every metabolite of the network could be a seed, which is not the usual use-case of the implementation, rather dedicated to reducing the size of *a priori* defined growth media.

Figure S7 describes in more depth the 10-solution results of the seed inference using the two tools. All 98 GSMNs solved by Seed2LP exhibit their entire set of solutions satisfying both FBA constraints and biomass reactants reachability. (Fig. S7a, e) All solutions inferred by COBRApy satisfy FBA constraints (Fig. S7a), but none satisfies NE constraint on biomass reactants. The size of seeds sets slightly larger for Seed2LP (Fig. S7c), and the average proportion of exchanged metabolites (according to the original model from BiGG) is overall similar across tools. Main differences between the tools relate to the proportion of reachable metabolites from seeds which is not a constraint in the MILP approach.

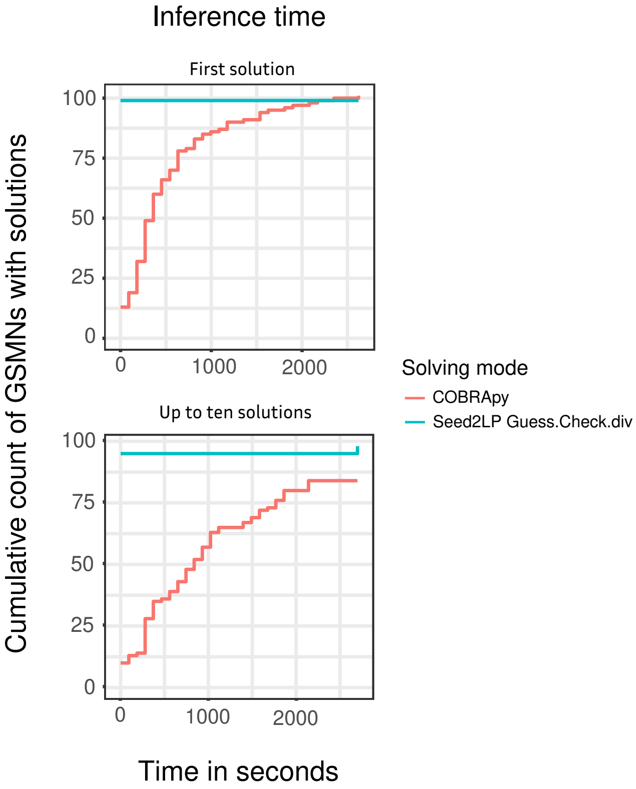

Figure S6: Solving time for seed inference with a MILP implementation from COBRApy and *Hybrid-*GC_{div} (targeting biomass reactants) mode. The cumulated number of GSMNs with one (top plot) or up to 10 (bottom plot) solutions over time and before a 45-minute timeout is illustrated.

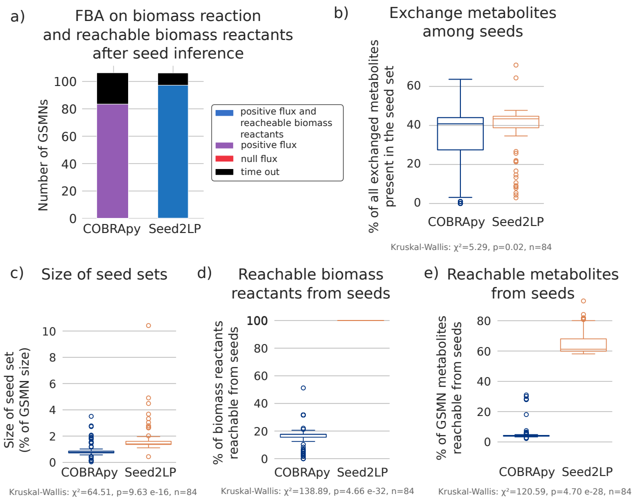

Figure S7: Seed inference with Seed2LP and COBRApy up to ten solutions. Exchanged reactions and corresponding transport reactions were created for all metabolites of the GSMNs prior using COBRApy `minimal_medium` function. a) Number of networks having all solutions validating FBA and ensuring biomass reactant reachability (blue) or all solutions validating FBA only (purple) or not (red) or in timeout (black). b) Proportion of all original exchange metabolites among the inferred seeds. c) Size of seed sets described as the proportion of the total number of metabolites per GSMN. d) Proportion (%) of reachable biomass reactants from the seeds out of the complete set of biomass reactants. e) Percentage of all GSMN metabolites reachable from the seeds. b), c), d), e): Distribution of the average proportions computed by GSMN if several solutions were obtained for a given GSMN; only results from the 84 GSMNs for which both COBRApy and Seed2LP find solutions are plotted.

9. Impact of NE flavours

We mentioned in the introduction that an alternative to NE takes into account self-regenerating cycles in an attempt to resemble, although qualitatively, the approach of FBA [18, 19]. An ASP-based implementation of seed solving with such an underlying model is available with the Precursor tool (`https://github.com/bioasp/precursor`). We compared Seed2LP with Precursor in order to assess the impact of NE flavour on qualitative seed inference.

Methods. We ran Precursor (commit a3acf85) on the 107 GSMNs of the BiGG database with a 45 minutes time limit, either in *Target* mode, i.e., setting up all biomass reactants as targets, or *Full Network* mode, i.e., setting up all GSMN metabolites as targets. We additionally tested the impact of Seed2LP network-model reconciliation prior to the Precursor run.

Results. We first refer to Precursor results with *a priori* reconciliation provided by Seed2LP (Fig. S8). At least one solution was obtained before timeout for all GSMNs in *Full Network* mode, and 11 of them held a positive biomass flux in FBA. Interestingly, those latter were the same ones as those holding a positive flux in the NetSeed experiment (Fig. S3), suggesting shared topological characteristics among them. Precursor raised solutions for all GSMNs in *Target* mode as well, although no one was FBA compliant (Fig. S8a). An interesting observation is the relatively small size of Precursor's seed sets with respect to the size of the network that arises from the consideration of self-regenerating cycles in GSMNs (Fig. S8b). Precursor generates sets of seeds ranging from 6 to 285 metabolites in *Full Network* mode, and 1 to 18 in *Target* mode whereas Seed2LP solutions contain between 6 and 59 seeds in *Target* mode and *Reasoning* method. Less solutions are found by Precursor than by Seed2LP: 82% of GSMNs have less than 8 solutions in 45 minutes in *Target* mode. Finally, we took a closer look at iCN718, as we analysed it further with Seed2LP. In *Full Network* mode, Precursor found a unique solution before timeout for this GSMN, consisting in 141 metabolites and enabling a positive flux in the biomass reaction. 246 solutions of size 1 (n = 245) or 2 (n = 1) seeds where found in *Target* mode, none satisfying FBA constraints. The impact of normalisation was null regarding the FBA results. It only slightly changed the number of solutions raised by Precursor for some GSMNs.

Overall, even if the network expansion flavour modelled in Seed2LP does not account for self-regenerating cycles, results from its reasoning modes (Fig. S3 for *Full Network* mode, Main Table 1 for *Target* mode) are more compatible with FBA constraints than Precursor.

10. Supplementary results on the iCN718 analysis

Table S1 provides solving details on the inference of seeds for the iCN718 GSMN, either in the 45-minute or 1000 solution set-up, or after 2000 solutions.

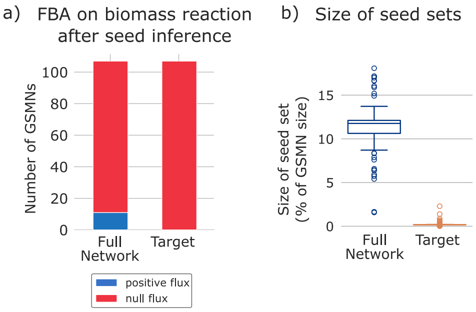

Figure S8: Results of Precursor seed inference. a) Number of GSMNs having all solutions validating FBA (blue) or none (red) or without any solution (black) in *Full Network* or *Target* search modes. b) Size of seed sets described as the proportion of the total number of metabolite per GSMN. Distribution of the average proportions computed by GSMN if several solutions were obtained for a given GSMN.

Test	Nb solutions	Nb rejected	Time	Union	Intersection
45-minutes TO or 1000 solutions	R: 1,000<br>F: 149<br>GC: 116<br>GCD: 111	R: NA<br>F: 799<br>GC: 801<br>GCD: 811	R: 6.209s<br>F: 45m<br>GC: 45m<br>GCD: 45m	R: 161<br>F: 67<br>GC: 98<br>GCD: 118	R: 1<br>F: 2<br>GC: 2<br>GCD: 0
2000 solutions	2,000	R: NA<br>F: 9,964<br>GC: 14,368<br>GCD: 9,273	R: 6.403s<br>F: ≤8d<br>GC: ≤8d<br>GCD: ≤8d	R: 199<br>F: 117<br>GC: 135<br>GCD: 149	R: 0<br>F: 1<br>GC: 2<br>GCD: 0

Table S1: Solving-associated details for seed inference in iCN718. First row depicts the results in the main benchmark presented in the manuscript (45 minute timeout or stop after 1000 computed solutions). Second row illustrates the results for computing 2000 solutions in each mode. The number of rejected models corresponds to solutions that were discarded because they did not validate FBA. Union and intersection describe the number of metabolites occurring in at least one solution and all solutions respectively. Abbreviations: R, *Reasoning*; F, *Hybrid-filter*; GC, *Hybrid-GC*; GCD, $\textit{Hybrid-GC}_{div}$; TO, Timeout; Nb, Number.

Figure S9 present the characteristics of seed solutions in the four inference modes. We observe that the number of exchanged metabolites covered by reasoning solutions is lower than for hybrid ones (Fig. S9a). The seed set sizes remain low in all four modes (Fig. S9b). 73% of all GSMN metabolites are reachable with sets of seeds in average, but small variation occurs depending on the inference mode (Fig. S9c).

Figure S10 presents the frequency of occurrence of seed metabolites across the 2000 solutions computed by the 4 inference modes of Seed2LP. We observe that exchanges metabolites ($n = 128$) as described in the original SBML model are more frequently present in seeds computed with hybrid modes, suggesting that this characteristic may be associated to solutions which validate FBA. Nonetheless, as it grasps the most diverse set of metabolites, the reasoning mode is the one that retrieves the most exchanges metabolites within its union (37.5%).

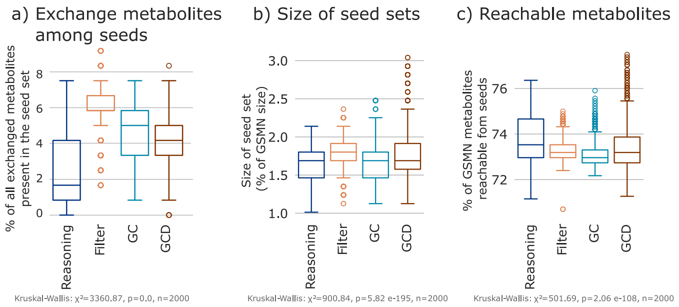

Figure S9: Characteristics of the 2000 solutions computed for iCN718: proportion of exchanged metabolites among seeds (a), size of seed sets with respect to the size of the GSMN (b), and proportion of reachable metabolites from the seeds (c), all according to the inference mode (*reasoning*, *Hybrid-lpx*, *Hybrid-filter*, *Hybrid-GC* or *Hybrid-GC$_{div}$*).

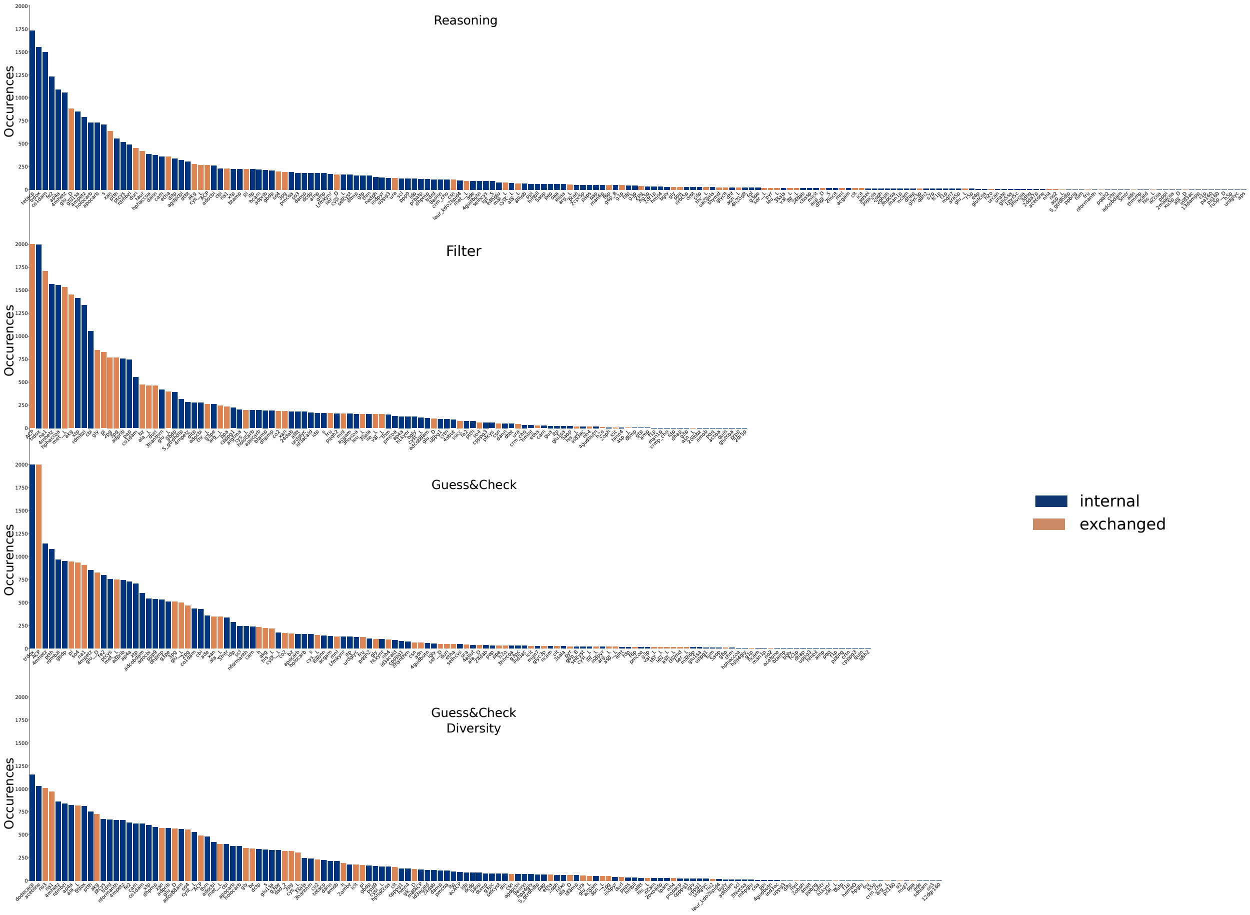

Figure S10: Occurrence of metabolites across the 2000 solutions according to the inference mode (*Reasoning*, *Hybrid-lpx*, *Hybrid-filter*, *Hybrid-GC* or *Hybrid-GC$_{div}$*). Orange metabolites are imported (exchanged metabolites) in the original model, whereas blue ones are internal metabolites.

We surveyed consequences of the differences between NE and FBA formalisms in an application to iCN718. The model from the database has a collection of exchange reactions depicting the growth medium and enabling a positive flux in the biomass reaction in FBA. Seed2LP can be run on the network without discarding the exchange reactions, in order to highlight compounds that are missing to ensure NE. Dismissing these exchange

reactions prior seed inference generates subset-minimal sets of seeds of size ranging from 9 to 19 (*Reasoning*, 1000 solutions) to 10 to 28 (*Hybrid solving modes*, 1000 solutions). Keeping the existing exchange reactions highlights the remaining seeds needed to ensure reachability of biomass reactants. We enumerated 5 solutions with the *Reasoning* mode to estimate the cost of reaching these metabolites, obtaining sets of seeds of size 5 to 6.

References

[1] Arnaud Belcour, Clémence Frioux, Méziane Aite, Anthony Bretaudeau, Falk Hildebrand, and Anne Siegel. Metage2Metabo, microbiota-scale metabolic complementarity for the identification of key species. *eLife*, 9:e61968, 2020. doi: 10.7554/elife.61968.

[2] Méziane Aite, Marie Chevallier, Clémence Frioux, Camille Trottier, Jeanne Got, María Paz Cortés, Sebastián N. Mendoza, Grégory Carrier, Olivier Dameron, Nicolas Guillaudeux, Mauricio Latorre, Nicolás Loira, Gabriel V. Markov, Alejandro Maass, and Anne Siegel. Traceability, reproducibility and wiki-exploration for “à-la-carte” reconstructions of genome-scale metabolic models. *PLOS Computational Biology*, 14(5):e1006146, 2018. ISSN 1553-734X. doi: 10.1371/journal.pcbi.1006146.

[3] Charles J Norsigian, Neha Pusarla, John Luke McConn, James T Yurkovich, Andreas Dräger, Bernhard O Palsson, and Zachary King. BiGG Models 2020: multi-strain genome-scale models and expansion across the phylogenetic tree. *Nucleic Acids Research*, 2019. ISSN 0305-1048. doi: 10.1093/nar/gkz1054.

[4] Elhanan Borenstein, Martin Kupiec, Marcus W. Feldman, and Eytan Ruppin. Large-scale reconstruction and phylogenetic analysis of metabolic environments. *Proceedings of the National Academy of Sciences*, 105(38):14482–14487, 2008. ISSN 0027-8424. doi: 10.1073/pnas.0806162105.

[5] Rogan Carr and Elhanan Borenstein. NetSeed: a network-based reverse-ecology tool for calculating the metabolic interface of an organism with its environment. *Bioinformatics*, 28(5):734–735, 2012. ISSN 1367-4803. doi: 10.1093/bioinformatics/btr721.

[6] Tony J. Lam, Moses Stamboulian, Wontack Han, and Yuzhen Ye. Model-based and phylogenetically adjusted quantification of metabolic interaction between microbial species. *PLOS Computational Biology*, 16(10):e1007951, 2020. ISSN 1553-734X. doi: 10.1371/journal.pcbi.1007951.

[7] Ali Ebrahim, Joshua A Lerman, Bernhard O Palsson, and Daniel R Hyduke. COBRApy: COnstraints-Based Reconstruction and Analysis for Python. *BMC Systems Biology*, 7(1):74, 2013. doi: 10.1186/1752-0509-7-74.

[8] Michael L. Waskom. seaborn: statistical data visualization. *Journal of Open Source Software*, 6(60):3021, 2021. doi: 10.21105/joss.03021. URL `https://doi.org/10.21105/joss.03021`.

[9] J. D. Hunter. Matplotlib: A 2d graphics environment. *Computing in Science & Engineering*, 9(3):90–95, 2007. doi: 10.1109/MCSE.2007.55.

[10] Wes McKinney. Data Structures for Statistical Computing in Python. In Stéfan van der Walt and Jarrod Millman, editors, *Proceedings of the 9th Python in Science Conference*, pages 56 – 61, 2010. doi: 10.25080/Majora-92bf1922-00a.

[11] Pauli Virtanen, Ralf Gommers, Travis E. Oliphant, Matt Haberland, Tyler Reddy, David Cournapeau, Evgeni Burovski, Pearu Peterson, Warren Weckesser, Jonathan Bright, Stéfan J. van der Walt, Matthew Brett, Joshua Wilson, K. Jarrod Millman, Nikolay Mayorov, Andrew R. J. Nelson, Eric Jones, Robert Kern, Eric Larson, C J Carey, İlhan Polat, Yu Feng, Eric W. Moore, Jake VanderPlas, Denis Laxalde, Josef Perktold, Robert Cimrman, Ian Henriksen, E. A. Quintero, Charles R. Harris, Anne M. Archibald, Antônio H. Ribeiro, Fabian Pedregosa, Paul van Mulbregt, and SciPy 1.0 Contributors. SciPy 1.0: Fundamental Algorithms for Scientific Computing in Python. *Nature Methods*, 17:261–272, 2020. doi: 10.1038/s41592-019-0686-2.

[12] William H. Kruskal and W. Allen Wallis. Use of ranks in one-criterion variance analysis. *Journal of the American Statistical Association*, 47(260):583–621, 1952. ISSN 1537-274X. doi: 10.1080/01621459.1952.10483441.

[13] Roie Levy, Rogan Carr, Anat Kreimer, Shiri Freilich, and Elhanan Borenstein. NetCooperate: a network-based tool for inferring host-microbe and microbe-microbe cooperation. *BMC Bioinformatics*, 16(1):164, 2015. doi: 10.1186/s12859-015-0588-y.

[14] Anat Kreimer, Adi Doron-Faigenboim, Elhanan Borenstein, and Shiri Freilich. NetCmpt: a network-based tool for calculating the metabolic competition between bacterial species. *Bioinformatics*, 28(16):2195–2197, 2012. ISSN 1367-4803. doi: 10.1093/bioinformatics/bts323.

[15] Aric A. Hagberg, Daniel A. Schult, and Pieter J. Swart. Exploring network structure, dynamics, and function using networkx. In Gaël Varoquaux, Travis Vaught, and Jarrod Millman, editors, *Proceedings of the 7th Python in Science Conference*, pages 11 – 15, Pasadena, CA USA, 2008. URL `http://conference.scipy.org/proceedings/SciPy2008/paper_2/`.

[16] Robert Tarjan. Depth-first search and linear graph algorithms. *SIAM Journal on Computing*, 1(2):146–160, 1972. doi: 10.1137/0201010. URL `https://doi.org/10.1137/0201010`.

[17] E. Nuutila and E. Soisalon-Soininen. On finding the strongly connected components in a directed graph. *Information Processing Letters*, 49:9–14, 1994. ISSN 1872-6119.

[18] Ludovic Cottret, Paulo Vieira Milreu, Vicente Acuña, Alberto Marchetti-Spaccamela, Fábio Viduani Martinez, Marie-France Sagot, and Leen Stougie. Enumerating Precursor Sets of Target Metabolites in

a Metabolic Network. In *Algorithms in Bioinformatics. WABI 2008. Lecture Notes in Computer Science*, volume 5251, pages 233–244, 2008. ISBN 9783540873600. doi: 10.1007/978-3-540-87361-7_20.

[19] Vicente Acuña, Paulo Vieira Milreu, Ludovic Cottret, Alberto Marchetti-Spaccamela, Leen Stougie, and Marie-France Sagot. Algorithms and complexity of enumerating minimal precursor sets in genome-wide metabolic networks. *Bioinformatics*, 28(19):2474–2483, 2012. ISSN 1367-4803. doi: 10.1093/bioinformatics/bts423.
